# Magnetic resonance imaging evaluation of spinal cord lesions: what can we find? - Part 1. Neoplastic, vascular, metabolic, and traumatic injuries

**DOI:** 10.1590/0100-3984.2020.0127

**Published:** 2021

**Authors:** Ronaldo Gonçalves Pereira, Bruno Niemeyer de Freitas Ribeiro, Thais Ribeiro Gomes Coutinho Pereira, Paulo Roberto Valle Bahia, Edson Marchiori

**Affiliations:** 1 Hospital Casa de Portugal / 3D Diagnóstico por Imagem, Rio de Janeiro, RJ, Brazil.; 2 Grupo Labs Fleury/RJ, Rio de Janeiro, RJ, Brazil.; 3 Instituto Estadual do Cérebro Paulo Niemeyer, Rio de Janeiro, RJ, Brazil.; 4 Universidade Federal do Rio de Janeiro (UFRJ), Rio de Janeiro, RJ, Brazil.

**Keywords:** Magnetic resonance imaging, Spinal cord injuries, Neoplasms, Metabolic diseases, Wounds and injuries/diagnostic imaging, Ressonância magnética, Traumatismos da medula espinal, Neoplasias, Doenças metabólicas, Ferimentos e lesões/diagnóstico por imagem

## Abstract

Diseases involving the spinal cord include a heterogeneous group of abnormalities, including those of inflammatory, infectious, neoplastic, vascular, metabolic, and traumatic origin. Making the clinical differentiation between different entities is often difficult, magnetic resonance imaging being the diagnostic method of choice. Although the neuroimaging findings are not pathognomonic, many are quite suggestive, and the radiologist can assist in the diagnosis and, consequently, in the therapeutic guidance. In this first part of our article, the objective is to review the magnetic resonance imaging findings of the main neoplastic, vascular, metabolic, and traumatic spinal cord injuries.

## Abstract (pt)

As doenças que envolvem a medula espinal incluem um grupo heterogêneo de anomalias, englobando causas inflamatórias, infecciosas, neoplásicas, vasculares, metabólicas e traumáticas. Muitas vezes a diferenciação clínica entre as diversas entidades é difícil, sendo a ressonância magnética a modalidade de escolha na investigação diagnóstica. Apesar de os achados de neuroimagem não serem patognomônicos, muitos são bastante sugestivos, podendo o radiologista auxiliar no diagnóstico e, consequentemente, na orientação terapêutica. O objetivo desta primeira parte do artigo é revisar os achados de ressonância magnética das principais lesões medulares neoplásicas, vasculares, metabólicas e traumáticas.

## INTRODUCTION

The spinal cord is the portion of the central nervous system that is within the vertebral canal, extending from the foramen magnum to the conus medullaris at the level of L1/L2, being surrounded by cerebrospinal fluid and contained by the thecal sac. Countless diseases can affect the spinal cord, leading to motor, sensory, and autonomic alterations, and magnetic resonance imaging (MRI) findings are essential for diagnostic elucidation and therapeutic orientation.

The evaluation of the nervous system by imaging methods has been the subject of a series of recent articles in the radiology literature of Brazil^**(1-5)**^. In this first part of our article, the objective is to review the MRI findings of the main neoplastic, vascular, metabolic, and traumatic spinal cord injuries.

## NEOPLASTIC CAUSES

### Astrocytoma

**Figure 1:**
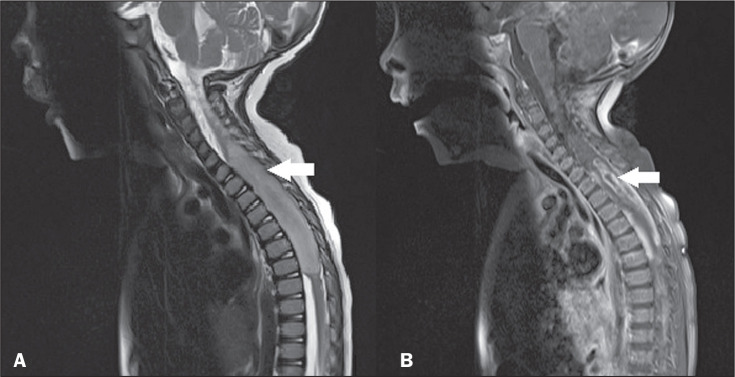
Astrocytoma. Sagittal MRI scans. A: T2-weighted sequence showing a spinal cord injury with a hyperintense signal, causing spinal cord expansion at the cervicothoracic junction (arrow). B: Contrast-enhanced T1-weighted sequence, showing predominantly peripheral irregular contrast enhancement (arrow).

Astrocytomas constitute the most common neoplastic cause of spinal lesions in children, being the second leading cause in adults and occurring more frequently in males. The site most commonly involved is the thoracic spine, followed by the cervical segment^**(6,7)**^. On MRI, these tumors are typically eccentric and have poorly defined margins, presenting a signal that is isointense or hypointense on T1-weighted images, and hypointense on contrast-enhanced T2-weighted images (Figure 1).

### Ependymoma

**Figure 2:**
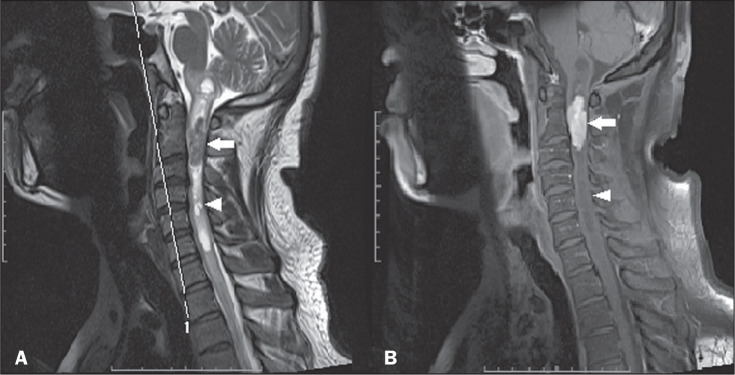
Ependymoma. Sagittal MRI scans. A: T2-weighted sequence showing an expansile lesion in the cervical spine (arrow), together with polar cysts (arrowhead). B: Contrast-enhanced T1-weighted sequence showing intense contrast enhancement (arrow), with no indication of contrast uptake by the polar cysts (arrowhead).

**Figure 3:**
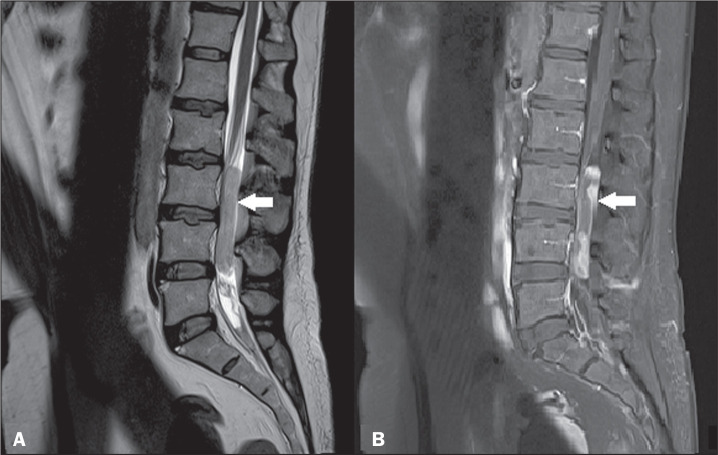
Ependymoma of the filum terminale. Sagittal MRI scans. A: T2-weighted sequence showing a hyperintense expansile lesion in the filum terminale (arrow). B: Contrast-enhanced T1-weighted sequence showing heterogeneous contrast enhancement (arrow).

Ependymomas constitute the most common neoplastic cause of spinal lesions in adults, especially in the fourth decade of life, and second most common cause in children, with a higher incidence in male patients and patients with type II neurofibromatosis, preferentially involving the cervical spine^**(7)**^. The tumor develops from the ependymal cells lining the central canal, which explains the fact that the symptoms are predominantly sensory. However, patients with a large ependymoma often present motor symptoms. On MRI, these tumors show a signal that is isointense or hypointense on T1-weighted images and hypointense, with contrast uptake, on contrast-enhanced T2-weighted images, whereas polar cysts (which are typically non-neoplastic) and edema of the surrounding bone marrow are common (Figure 2). The “mushroom cap” sign (a hypointense signal in the margins of the lesion, secondary to previous hemorrhages, on T2-weighted images) may be present. Calcification is uncommon. The myxopapillary variant occurs almost exclusively in the conus medullaris and filum terminale, where the tumor is most commonly located (Figure 3). In some cases, an ependymoma leads to subarachnoid hemorrhage.

### Ganglioglioma

**Figure 4:**
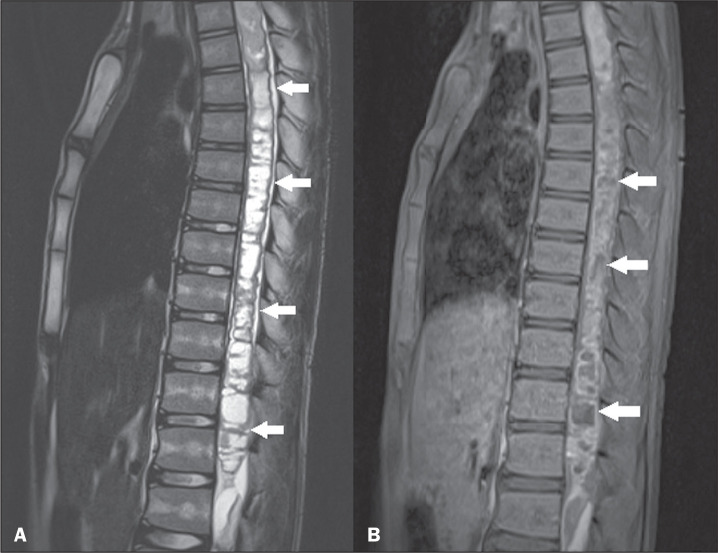
Ganglioglioma. Sagittal MRI scans. A: T2-weighted sequence showing a hyperintense signal in the long segments of the thoracic and lumbar spine (arrows). B: Contrast-enhanced T1-weighted sequence showing heterogeneous contrast enhancement (arrows).

A ganglioglioma is a rare spinal cord tumor that is most common in children and is most often found in the cervical spine, the thoracic spine being the second most common location. Gangliogliomas rarely affect the conus medullaris. Classically, they involve long segments of the spine, extending across more than eight vertebral segments. Most of the segments contain cysts. On MRI, the signal is hypointense on T1-weighted images and hyperintense on contrast-enhanced T2-weighted images, with heterogeneous contrast enhancement (Figure 4). Bone changes, including scoliosis and remodeling, are much more common in patients with gangliogliomas than in those with other types of tumors^**(8)**^.

### Hemangioblastoma

**Figure 5:**
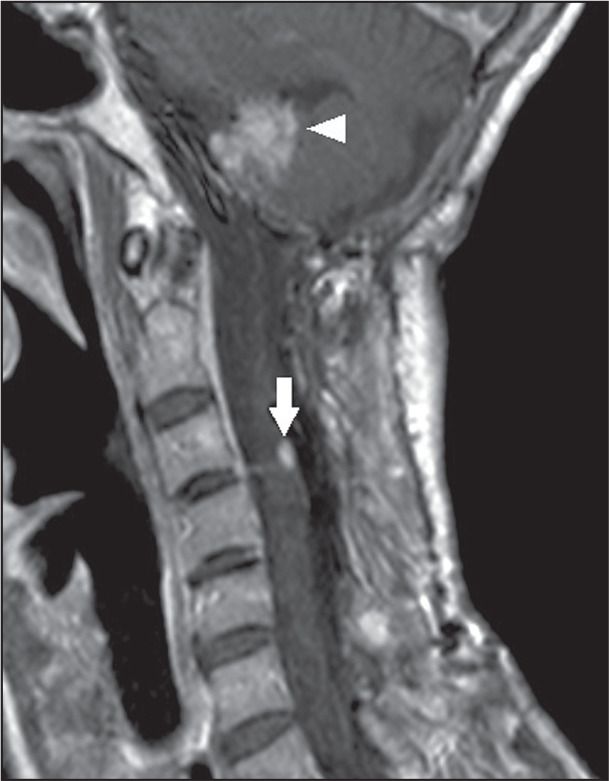
Hemangioblastoma. Sagittal MRI scan of a patient with Von Hippel-Lindau syndrome. Contrast-enhanced T1 sequence showing an expansile lesion, with intense contrast enhancement, in the posterior region of the cervical spine (arrow) and another hemangioblastoma in the cerebellum (arrowhead).

A hemangioblastoma is a benign, proliferative vascular lesion found commonly in the cerebellum and rarely in the spine. Most cases occur in males, around the fourth decade of life, and are commonly related to the Von Hippel-Lindau syndrome. The clinical presentation includes lower back or chest pain, with signs of radiculopathy and myelopathy. The most affected region is the thoracic spine, followed by the cervical spine. On MRI, the lesion typically presents as a hyperintense nodule on contrast-enhanced T2-weighted images, with pronounced contrast enhancement, and may show unrestricted diffusion on diffusion-weighted imaging, the “mushroom cap” sign, and perilesional edema^**(9)**^, as depicted in Figure 5. Solid-cystic lesions may be seen, although they are much less common in the spine than in the cerebellum.

### Metastases

**Figure 6:**
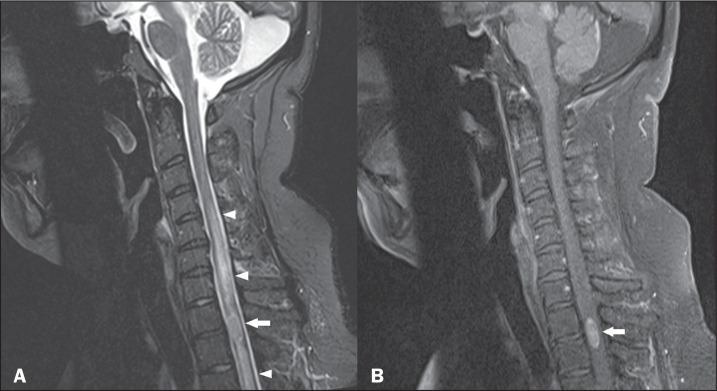
Spinal cord metastasis of clear cell renal carcinoma. Sagittal MRI scans. A: Short-tau inversion-recovery sequence showing a spinal cord lesion (arrow) with perilesional edema (arrowheads). B: Contrast-enhanced T1-weighted sequence showing marked contrast enhancement (arrow).

Metastases to the spinal cord are rare, being more common in patients with advanced-stage cancer. In order of frequency, the sites involved are the cervical, thoracic, and lumbar spine. Metastases to the spinal cord are typically solitary. The most common primary site is the lung, followed by the breast, although any tumor can metastasize to the spinal cord. On MRI, metastases can result in the expansion of spinal segments, with intense, homogeneous contrast enhancement, a common finding being perilesional edema that is, at times, disproportionate to the size of the lesion (Figure 6). Unlike primary spinal cord neoplasms, accompanying cysts are rare^**(7)**^.

## METABOLIC CAUSES

### Friedreich ataxia

Friedreich ataxia is a rare recessive autosomal hereditary disease, less well known than genetic cerebellar ataxia, with symptom onset around the second decade of life, and may also be accompanied by scoliosis, foot deformity, and hypertrophic cardiomyopathy. On MRI, the spinal cord may show a reduction in its anteroposterior diameter, together with changes in signal intensity in its posterior and lateral columns^**(10)**^.

### Vitamin B12 deficiency

**Figure 7:**
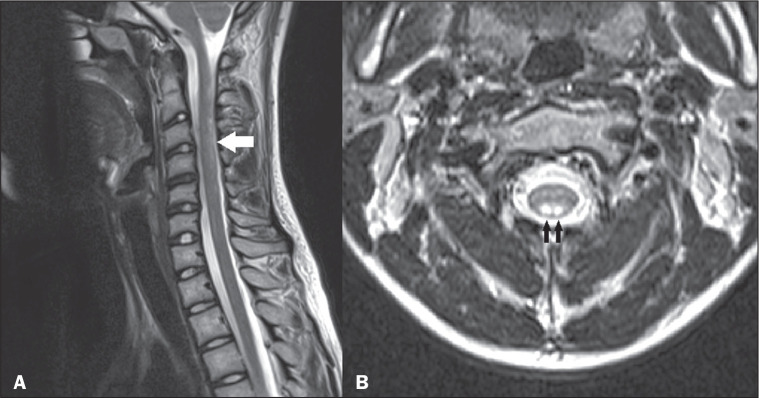
Vitamin B12 deficiency. Sagittal and axial T2-weighted MRI sequences (A and B, respectively), showing a hyperintense signal in the posterior region of the cervical spine (arrows).

Vitamin B12 deficiency is characterized by subacute combined degeneration of the spinal cord, presenting as a loss of proprioception, together with perceived vibration in the hands and feet, and may evolve to ataxia and dementia. On MRI, a symmetric bilateral hyperintense signal is observed in the posterior region of the spinal cord, typically affecting the cervical and upper thoracic regions, with ascending or descending progression (Figure 7). These changes may regress after correction of the vitamin deficiency^**(6,7)**^.

### Copper deficiency

Copper deficiency is a rare condition that causes progressive myelopathy, with fatigue, sensory ataxia, and a spastic gait, beginning at the extremities and progressing toward the waist. The time from the onset of the neurological symptoms to the diagnosis of myelopathy ranges from two months to years, and the clinical evolution of patients with myelopathy secondary to copper deficiency includes combined subacute degeneration similar to that seen in vitamin B12 deficiency. On MRI, a hyperintense signal can be seen on T2-weighted images, the incidence being higher in the posterior portion of the cervicothoracic spine, and there may be regression after copper replacement^**(11)**^.

## VASCULAR/TRAUMATIC CAUSES

### Spinal cord infarction

**Figure 8:**
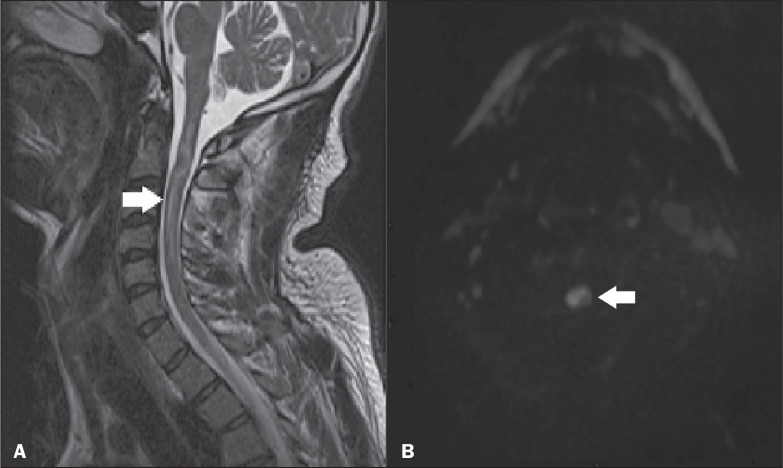
Spinal cord infarction after radiofrequency facet joint denervation. Sagittal short-tau inversion-recovery MRI sequence (A) and axial diffusion-weighted MRI sequence (B), showing a hyperintense signal (arrow in A) and restricted diffusion (arrow in B).

Spinal cord infarction is a rare condition with a poor prognosis and a clinical presentation that is dependent on the location and extent of involvement^**(12)**^. In children, it is commonly related to cardiac malformations and trauma, whereas the main causes in adults are cardiovascular diseases, such as hypotension and dissection. Spinal cord infarction typically affects the anterior aspect of the spinal cord, secondary to occlusion of the anterior spinal artery^**(6,7)**^. On MRI, a hyperintense signal can be seen on T2-weighted images, together with restricted diffusion on diffusion-weighted imaging, and a bilateral, symmetrical signal hyperintensity can be seen on T2-weighted images, in the gray matter of the anterior horns of the spinal cord (Figure 8), although this latter finding is also seen in other diseases, such as compressive myelopathy and polio-like syndromes.

### Spinal trauma

**Figure 9:**
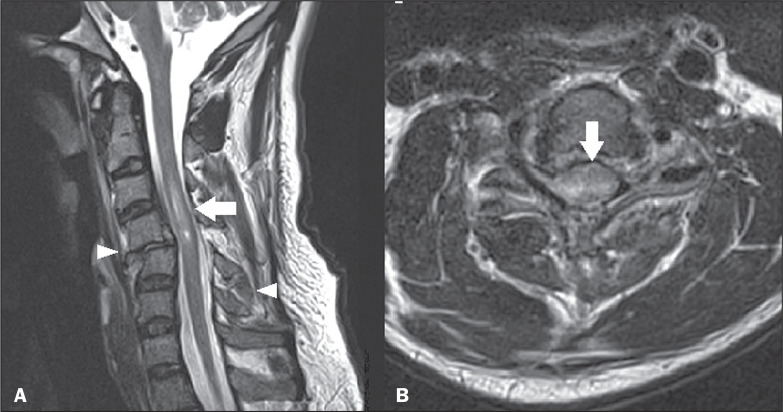
Cervical spine trauma. Sagittal and axial T2-weighted MRI sequences (A and B, respectively), showing a hyperintense signal and a volume increase in the cervical region (arrows). Similar changes can be seen in the cervical spine (arrowheads).

Spinal trauma is a common cause of acute myelopathy. In younger individuals, spinal trauma is typically due to motor vehicle accidents, most often affecting the cervical spine, whereas spinal trauma in the elderly is typically due to a fall, most often affecting the thoracolumbar spine^**(13)**^. The most common imaging patterns in cervical spine trauma are edema, contusion, hemorrhage, extrinsic compression, and spinal cord transection. On MRI, spinal cord edema presents as intumescence and a focal hyperintense signal in T2-weighted sequences, whereas contusions/hemorrhages present focal hyperintense signals in T1-weighted sequences, together with a hypointense signal on susceptibility-weighted imaging (Figure 9). Spinal cord transection is seen as spinal cord discontinuity, best observed in sagittal sequences.

### Cavernoma

**Figure 10:**
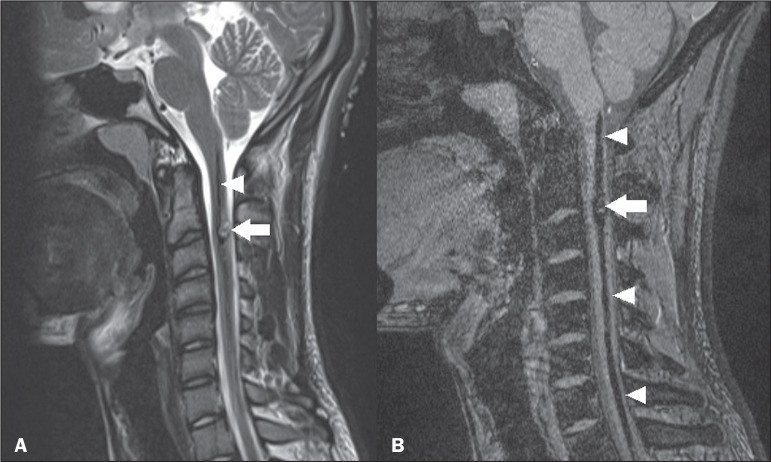
Cavernoma. Sagittal MRI scans. T2-weighted sequence (A) and susceptibility-weighted sequence (B), showing a heterogeneous spinal cord lesion, characterized by central hyperintensity and peripheral hypointensity in the T2-weighted sequence (arrow) and marked hypointensity in the susceptibility-weighted sequence (arrow). Note the bleeding from the lesion within the spinal canal, as evidenced by the linear hypointensity, better isolated in the susceptibility-weighted sequence (arrowheads in A and B).

A cavernoma is a vascular malformation with a slight predominance in females in the fourth decade of life, the most affected site being the thoracic spine, followed by the cervical spine. On MRI, a cavernoma is characterized by a lesion with a hyperintense central signal and a hypointense halo on T2-weighted images, with a markedly hypointense signal on susceptibility-weighted imaging, without pronounced uptake by the contrast medium^**(6,7,14)**^, as illustrated in Figure 10.

### Nontraumatic, sports-related vascular myelopathy

Nontraumatic, sports-related vascular myelopathy is rare, primarily affecting children and young adults. It is characterized by back pain in general or low back pain, as well as acute onset paraparesis. Although the pathophysiology is uncertain, it is believed to be secondary to spinal cord ischemia triggered by hyperextension of the spine for prolonged periods, being classically described in individuals who surf. On MRI, the typical presentation is of a long longitudinal lesion extending from the thoracic region to the conus medullaris, affecting the central portion of the spine and characterized by a hypointense signal on T2-weighted images, restricted diffusion on diffusion-weighted imaging, and the absence of contrast enhancement. Outcomes range from total recovery to persistent paraplegia^**(7,15)**^.

## CONCLUSION

In view of the aspects described above, it is obvious that spinal cord lesions pose a challenge for clinicians and radiologists. However, neuroimaging findings, when taken together with clinical and biochemical data, may facilitate the diagnosis and guide the treatment. Therefore, radiologists should be prepared to interpret such findings.
